# The Spleen Tyrosine Kinase Inhibitor, Entospletinib (GS-9973) Restores Chemosensitivity in Lung Cancer Cells by Modulating ABCG2-mediated Multidrug Resistance

**DOI:** 10.7150/ijbs.61229

**Published:** 2021-06-22

**Authors:** Silpa Narayanan, Zhuo-Xun Wu, Jing-Quan Wang, Hansu Ma, Nikita Acharekar, Jagadish Koya, Sabesan Yoganathan, Shuo Fang, Zhe-Sheng Chen, Yihang Pan

**Affiliations:** 1 Department of Pharmaceutical Sciences, College of Pharmacy and Health Sciences, St. John's University, Queens, NY, 11439, USA.; 2 Precision Medicine Center, The Seventh Affiliated Hospital, Sun Yat-Sen University, Shenzhen 518107, PR China.; 3 Department of Oncology, The Seventh Affiliated Hospital, Sun Yat-Sen University, Shenzhen, 518107, PR China.

**Keywords:** Entospletinib, Syk, MDR, reversal effect, ATP binding cassette transporter, ABCG2

## Abstract

Tyrosine kinase inhibitors (TKIs) are important in managing lymphoid malignancies by targeting B-cell receptor signaling pathways. Entospletinib (GS-9973) is an oral, selective inhibitor of spleen tyrosine kinase (Syk), currently in the phase II clinical trials for the treatment of chronic lymphocytic leukemia. Syk is abundantly present in the cells of hematopoietic lineage that mediates cell proliferation, differentiation, and adhesion. In this current study, we evaluated the efficacy of GS-9973 to overcome multidrug resistance (MDR) due to the overexpression of the ABCG2 transporter in the non-small cell lung cancer (NSCLC) cell line, NCI-H460/MX20. *In vitro*, 3 μM of GS-9973 reversed the drug resistance of NCI-H460/MX20 cell line to mitoxantrone or doxorubicin. GS-9973, at 3 μM reverses ABCG2-mediated MDR by blocking ABCG2 efflux activity and downregulating ABCG2 expression at the protein level but did not alter the ABCG2 mRNA expression and subcellular localization of the ABCG2 protein compared to drug-resistant cells incubated with the vehicle. GS-9973 produced a moderate concentration-dependent increase in the ATPase activity of ABCG2 (EC_50_ = 0.42 µM) and molecular docking data indicated that GS-9973 had a high affinity (-10.226 kcal/mol) for the substrate-binding site of ABCG2. Finally, HPLC analysis proved that the intracellular concentration of GS-9973 is not significantly different in both parental and resistant cell lines. In conclusion, our study suggests that *in vitro*, GS-9973 in combination with certain anticancer drugs, represent a strategy to overcome ABCG2-mediated MDR cancers.

## Introduction

Lung cancer is the leading source of cancer associated casualties globally, with an estimated 2.21 million cases in the year 2020 and around 1.79 million deaths in 2020, and non-small cell lung cancer (NSCLC) accounts for up to 85% of the lung cancer cases all around the world 1. The 5-year survival rate of NSCLC patients from the time of diagnosis was found to be around 15% because more than 50% of NSCLC cases are diagnosed at metastatic conditions (stage IV) 2-4. Regardless of achieving substantial advancements in the treatment of metastatic or locally advanced NSCLC, improving the quality of life in NSCLC patients has been a major challenge 4. Treatment modalities involving surgery, radiation therapy, and chemotherapy are considered essential to treat NSCLC patients but tumor heterogenicity and multidrug resistance (MDR) remain to be a primary obstacle by limiting chemotherapeutic efficacy leading to increased cases of relapse and mortality in NSCLC patients 5-10. MDR or chemoresistance characterizes a phenomenon where cells exhibit resistance to drugs that are pharmacologically and structurally distinct 11-15. MDR is orchestrated via different mechanisms 16 among which, adenosine triphosphate binding cassette (ABC) transporters play a substantial role by extruding antineoplastic drugs and cytotoxic agents 14,17-19. ABC transporter superfamily embodies a ubiquitous range of comprehensive membrane proteins which are classified into seven subfamilies from ABCA to ABCG and are responsible for critical physiological and pharmacological contributions 20-22. ABCG2 was initially identified in MCF-7 breast cancer cells giving it the nomenclature, breast cancer resistance protein (BCRP) 23,24. ABCG2 has been widely reported to be a principal cause of MDR in various cancers via extruding various antineoplastic drugs such as tyrosine kinase inhibitors (TKIs), topoisomerase inhibitors, anthracyclines, etc. 25-29.

Platinum-based chemotherapy with or without bevacizumab is considered to be the first line of treatment in advanced NSCLC patients lacking targetable tumor-specific mutations 30. Considering the oncogene mutations and other factors in NSCLC patients, various targeted therapies have been developed such as EGFR specific TKIs targeting EGFR specific mutations, anaplastic lymphoma kinase (ALK) inhibitor targeting ALK-positive patients, and immune checkpoint blockers which substantially improved the quality of life in NSCLC patients over the past 20 years 31-33. Expression of ABCG2 is associated with a decrease in response to therapy and overall survival in NSCLC patients 3,8. Several studies have established that cancer stem-like cell (CSC) population in cancer expressing ABCG2 limits the efficacy of chemotherapy and is responsible for the re-emergence of tumors during the period of relapse in various cancer patients 34-36. Studies conducted by Hang et al and Li et al have determined that CSC expressing CD133 and ABCG2 have decreased overall survival, chemotherapeutic response rates, and increased the rate of relapses in NSCLC patients 37,38. *In vitro* studies have also demonstrated that several TKIs like imatinib, gefitinib, and nilotinib are substrates of ABCG2 39,40 or inhibitors of ABCG2 in some studies 41-43. Due to its potential role in regulating MDR and its association with NSCLC as a prognostic marker, there is a serious need to identify potential drugs that can resensitize ABCG2-mediated drug resistance.

Previous *in vitro* studies from our lab have shown some of the selective TKIs can sensitize ABCG2-mediated cancer resistance in various cancer scenarios 44-46. Spleen tyrosine kinase (Syk) is a cytoplasmic tyrosine kinase expressed ubiquitously in hematopoietic cells and in other cell types 47. Activation of Syk mediates the activation of B cells and T cells by eliciting the interaction between the T cell and B cell receptors 48. Syk signaling is associated with different biological responses comprising cellular proliferation, differentiation, function, development, and adhesion 49. Entospletinib (GS-9973) is a novel selective Syk inhibitor that is being evaluated for its efficacy in treating hematopoietic malignancies like chronic lymphocytic leukemia (CLL), mantle cell lymphomas, acute myeloid leukemia (AML), etc. 49-51. In current study, we tried to investigate the efficacy of GS-9973 in antagonizing the ABCG2-mediated chemoresistance in NSCLC cell lines.

## Materials and methods

### Chemicals

GS-9973 (Entospletinib) (Figure 4A) was kindly supplied by Chemietek (Indianapolis, IN). Mitoxantrone was bought from Enzo Sciences (Farmingdale, NY) and doxorubicin was obtained from Medkoo Biosciences (Morrisville, NC). Cisplatin, verapamil, vincristine, 3-(4,5-dimethylthiazol-yl)-2,5-diphenyltetrazolium bromide (MTT), and dimethyl sulfoxide (DMSO) were obtained from Sigma Chemical (St. Louis, MO). The cell culture medium, Dulbecco's modified Eagle's medium (DMEM), trypsin and penicillin/streptomycin (P/S) were purchased from Corning Life sciences (Manassas, VA). Ko143 was bought from Enzo Life Sciences (Farmingdale, NY) and [^3^H]-mitoxantrone (4 Ci/mmol) was acquired from Moravek Biochemicals, Inc (Brea, CA). ABCG2 (D752K) and GAPDH (D16H1) selective monoclonal antibodies and secondary anti-rabbit antibody linked with HRP (7074S) were procured from Cell Signaling Technologies (Danvers, MA). Alexa fluor conjugated secondary antibody was purchased from Molecular Probes (Eugene, OR). Trizol reagent was obtained from Invitrogen Life Technologies (Carlsbad, CA). The ABCG2 and GAPDH TaqMan gene expression kits and superscript IV reverse transcription kit were obtained from Fisher Scientific (Waltham, MA).

### Equipment

The AccuSkan GO microplate reader was procured from Thermo Fisher (Thermo Fisher Scientific, Finland) and the TRI-CARB1 1900CA liquid scintillation analyzer was obtained from Packard Instrument Company, Inc (Downers Grove, IL).

### Cell lines

NCI-H460, an NSCLC cell line, and mitoxantrone-selected ABCG2 overexpressing drug-resistant NCI-H460/MX20 cells were selected for this study. NCI-H460/MX20 cell line was established by culturing the sensitive/parental NSCLC cell line, NCI-H460 with the anticancer drug, mitoxantrone up to a concentration of 20 nM 52. HEK293/pcDNA3.1 (human embryonic kidney cell line transfected with empty vector), HEK293 cells transfected with the ABCG2 cDNA, HEK293/R482 (wild-type) and HEK293/R482G and HEK293/R482T (2 variants) cells were grown in G418 (geneticin, an aminoglycoside antibiotic) at a concentration of 2 mg/ml following transfection of HEK293/pcDNA3.1 enclosed with full-length ABCG2 cDNA coding with arginine (R), glycine (G) or threonine (T) at position 482 53. NCI-H460/MX20 has characteristics similar to that *in vivo* but multiple factors that can cause MDR other than overexpression of the ABCG2 transporter 54 and the role of ABCG2-mediated resistance can be confirmed by the use of transfected cell lines, although these cells are non-cancerous. All the cell lines including SW620 (parental) and SW620/AD300 colon cancer cells (overexpressing ABCB1 transporter) were cultured in DMEM medium supplemented with 10% FBS and 1% penicillin and streptomycin, in 5% CO_2_ at 37 °C. SW620/AD300 cells were established by culturing them in gradually increasing concentrations of doxorubicin. The HEK293/pcDNA3.1 cell line was transfected with the DNA coding for the ABCC1 transporter to generate the HEK293/ABCC1 cell line 55,56. Cells were cultured as a monolayer and were maintained in regular drug-free medium for at least two weeks prior to conduct the experiments.

### MTT assay for cytotoxicity determination and the reversal experiments

The MTT cytotoxicity assay was performed in a 96 well plate to determine the concentration of GS-9973 to be used in experiments with the parental and drug-resistant cell lines. A seeding density of 4×10^3^ cells/well was used for the entirety of cell lines. The absorbance was obtained using a spectrophotometer, set at 570 nm as previously described 57-60. The concentrations at which around 85% of parental and resistant cells survived (i.e., 1 and 3 µM) were used for the reversal experiments.

For the reversal experiments, parental NCI-H460 and drug-resistant NCI-H460/MX20 cells were treated for 2 h with 1 and 3 µM of GS-9973, 3 µM of Ko143, a known ABCG2 inhibitor. Following incubation, an anticancer agent, mitoxantrone, doxorubicin, cisplatin, or vincristine, was added at different concentrations (20 µl/well) into the designated wells. The MTT assay was performed after 72 h of incubation by recording the absorbance at 570 nm. The resistance folds were computed by dividing the IC_50_ value in resistant cells, with or without the drug (GS-9973 or Ko143), by the IC_50_ value of the parental cells.

### [^3^H]- Mitoxantrone accumulation and efflux assay

The intracellular accumulation and efflux activity of the ABCG2 transporter is estimated by incubating NCI-H460 and NCI-H460/MX20 cancer cells in the presence or absence of 1 and 3 µM of GS-9973 or Ko143 (3 µM) for 2 h at 37 °C followed by treating with 0.01 µM of [^3^H]-mitoxantrone (at 0, 30, 60 and 120 min) for 2 h as previously described 61. The radioactivity was quantified using a scintillation counter.

### Western blot analysis

Western blot analysis was carried out to detect the expression level of ABCG2 protein. NCI-H460 and NCI-H460/MX20 cells were cultured and seeded in T25 flasks (1 million cells/flask) and the next day, 3 µM of GS-9973 was added and incubated for different time points, 24, 48 and 72 h. Cell lysates were prepared by adding lysis buffer (25 mM Tris-HCl (pH 7.6), 150 mM NaCl, 1% Triton X-100, 1% sodium deoxycholate, 0.1% SDS) to cells overexpressing ABCG2. The ABCG2 protein expression assay was conducted using Western blotting as previously described 62. The samples were incubated with primary antibodies, ABCG2 (D5V2K) and GAPDH (D16H1) (1:1000) overnight at 4°C and then incubated with horse radish peroxidase-conjugated secondary antibody (7074S) (1:1000) for 2 h at room temperature. The reaction was visualized by enhanced chemiluminescence detection reagents (Amersham, NJ) using the manufacturer's protocol.

### mRNA expression

NCI-H460 and NCI-H460/MX20 cancer cells were incubated with 3 μM of GS-9973 for 24, 48 and 72 h and total RNA was extracted using the RNA extraction trizol reagent as previously described 63. RNA was quantified using Nanodrop and RNA samples with an A260/280 ratio in the range of 1.8 to 2.0 were subjected to reverse transcription and the cDNAs formed (by superscript IV reverse transcription kit) were used for quantitative PCR. This analysis was performed using the ABCG2 and GAPDH TaqMan gene expression assay kits. The PCR data were quantitated using the ∆∆Ct method and presented as relative - fold of mRNA expression.

### Immunofluorescence

Immunofluorescence assay was performed to evaluate whether the reversal activity of GS-9973 was involved in the subcellular localization of the ABCG2 membrane protein. For the immunofluorescence analysis, the parental and the drug-resistant cells were seeded in a 24 well plate at a density of 10,000 cells/well and treated with or without the vehicle or 3 µM of GS-9973 for different time points, 24, 48, and 72 h. Briefly, washing was done thrice with PBS, followed by fixing for 15 min in 4% paraformaldehyde, and permeabilized using 0.1% Triton X-100 for 15 min and subsequently blocking with 6% BSA. The ABCG2 transporter was distinguished by an anti-ABCG2 monoclonal antibody and then accompanied by Alexa Fluor 488 conjugated secondary antibody. Later, nuclei were counterstained with DAPI. The images were taken using an EVOS FL Autofluorescence microscope (Thermo Scientific, Waltham, MA). The study was conducted independently in triplicates.

### ATPase assay

The ABCG2 ATPase activity was carried out using PREDEASY ATPase Kits (TEBU-BIOnv, Boechout, Belgium) as mentioned previously 64. For the ATPase assay, 10 μg membrane with ABCG2 was incubated in the assay buffer. Then the membrane vesicles were incubated in GS-9973 for 3 min. An addition of 5 mM Mg-ATP activated the ATP hydrolysis. The reaction was terminated by adding 5% SDS solution after incubated for 20 min at 37 °C. The inorganic phosphate (Pi) was measured at 880 nm using a spectrophotometer.

### Molecular docking of GS-9973 with the human ABCG2 model

Docking experiments were performed on a Mac Pro 6-core Intel Xenon E5 processor with Macintosh Operating System (OS Sierra) using the Maestro v12. 3. 012 software (Schrödinger, LLC, New York, NY, USA, 2019) software. Lig-prep was used for GS-9973 ligand preparation 65. The human model of ABCG2 was imported from the Protein data bank. Protein Preparation Wizard was used for protein preparation. The grid was generated by selecting residues at 20 Å length from bound inhibitors in the protein (6ETI) 66. The residues selected were: 570, 571, 578, 624, 628, 631, 632, 635, 636, 639, 640. Extra Precision docking was performed with a maximum of 10 poses 67.

### HPLC analysis

NCI-H460 and NCI-H460/MX20 cells were seeded in a 6-well-plate at a density of 2 × 10^5^ cells per well and cultured for 2 days, media was replaced with plain media (DMEM without FBS), and was treated with 10 µM of GS-9973 for 2 h. Washing was done with PBS and 0.5% Sodium dodecyl sulfate and acetonitrile were added to the plate to lyse the cells for the extraction of the drug. The lysates were obtained and centrifuged at a speed of 14,000 rpm for 10 min and the supernatant formed was used to measure the intracellular concentration of GS-9973 by HPLC. HPLC was performed using the Agilent Technologies instrument (Prep218 pump and Prostar 325 detector) and monitored using a dual-wavelength detector (250 and 258 nm). The eluents used were, water (Solvent A) and acetonitrile (Solvent B), both solvents supplemented with 0.1% formic acid to maintain buffer capacity. Purification was done using a C8 analytical column (Agilent Eclipse Plus 3.5 mm, 4.6 X 150 mm). Flow rate: 1 ml/min. Isocratic solvent system: 60:40 water: acetonitrile and the run time was 15 min. The standard curve was plotted using different concentrations of GS-9973 were used (8 ng/ml, 4 ng/ml, 2 ng/ml, 1 ng/ml) against area under the curve (AUC).

### Statistical analysis

All experiments were repeated in triplicates and the data was analyzed using GraphPad Prism (version 8). The *a priori* significance level was *p* < 0.05 and the results were evaluated with one-way or two-way ANOVA and *post hoc* analysis was conducted using Dunnett's *post hoc* test.

## Results

### GS-9973 sensitizes ABCG2 overexpressing cells to anticancer drugs

**Figure 1:**
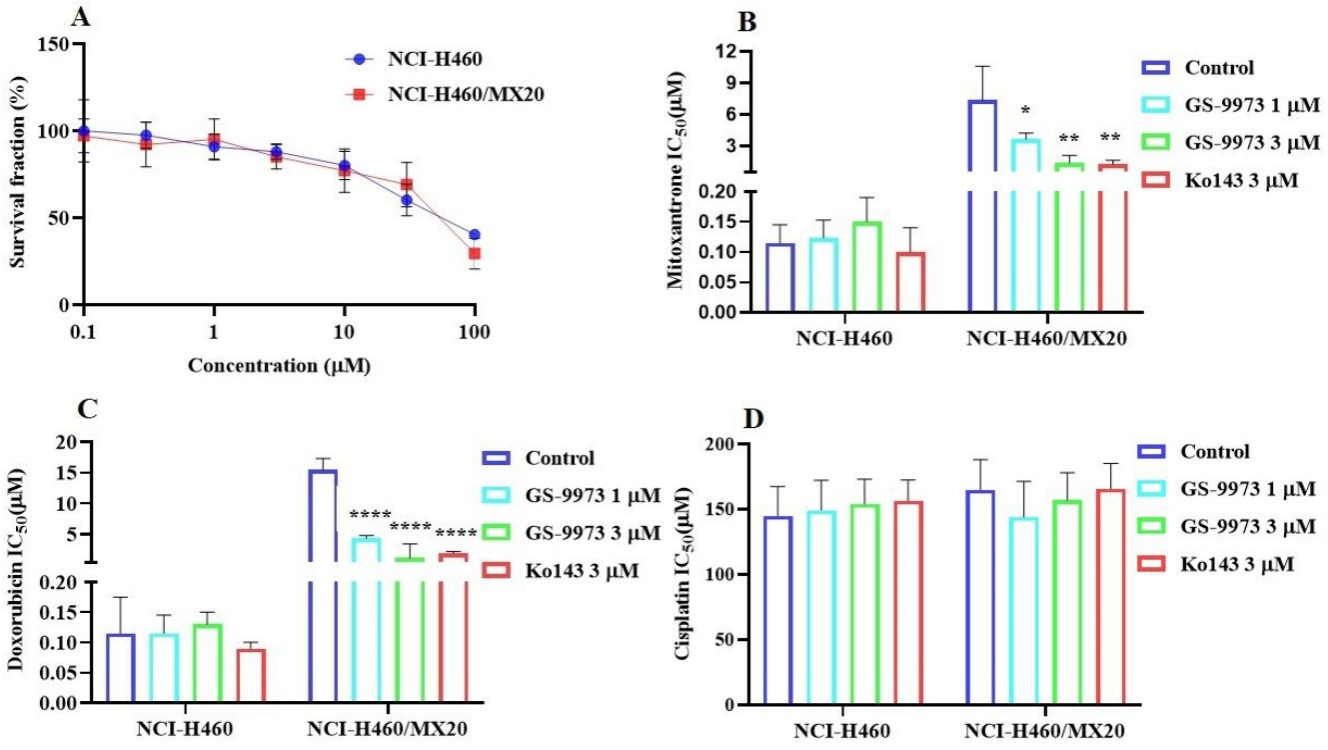
** The effect of GS-9973 in parental NCI-H460 and ABCG2-overepxressing NCI-H460/MX20 cancer cell lines. (A)** Th**e** survival fraction (%) was determined following incubation with 0.1-100 µM of GS-9973 for 72 h in NCI-H460 (blue) and NCI-H460/MX20 (red) cell lines and IC_50_ values of **(B)** mitoxantrone, **(C)** doxorubicin and **(D)** cisplatin in parental NCI-H460 and drug-selected ABCG2 overexpressing NCI-H460/MX20 cancer cells with or without GS-9973. All data are shown as mean ± SD and represents three independent experiments. *p ≤ 0.05, **p ≤ 0.01 and ****p < 0.0001 compared to the control group.

**Figure 2:**
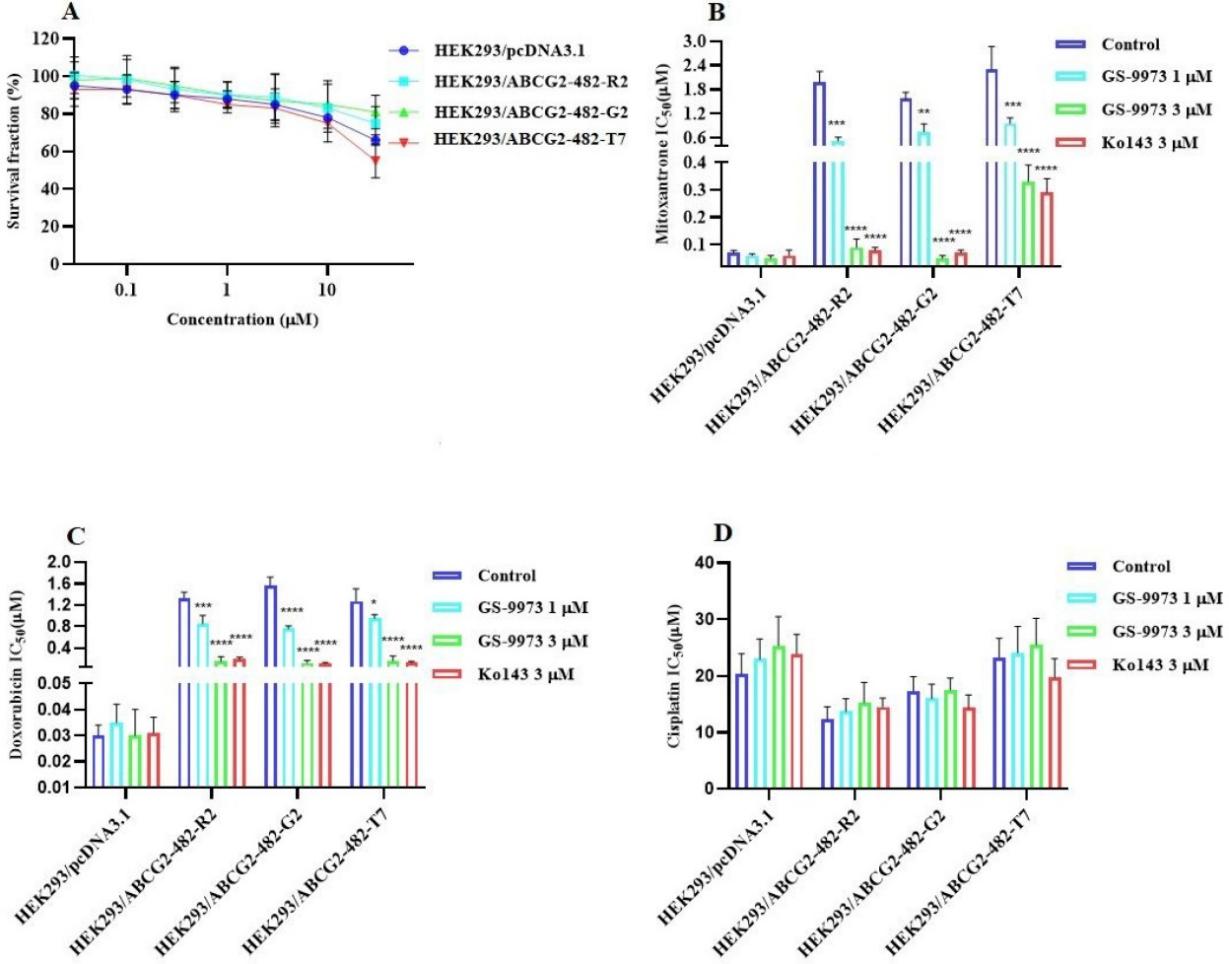
** The effect of GS-9973 in HEK293 cells transfected with the gene coding for the ABCG2 transporter. (A)** The survival fraction (%) for HEK293/pcDNA3.1 (empty DNA vector control), HEK293/ABCG2-482-R2, HEK293/ABCG2-482-G2 and HEK293/ABCG2-482-T7 cell lines was determined following incubation with 0.03-30 µM of GS-9973 for 72 h. The IC_50_ values of **(B)** mitoxantrone, **(C)** doxorubicin, and **(D)** cisplatin in HEK293/pcDNA3.1 (empty DNA vector control), HEK293/ABCG2-482-R2, HEK293/ABCG2-482-G2 and HEK293/ABCG2-482-T7 cell lines. Data are exhibited as mean ± SD and acquired from at least three independent experiments. *p ≤ 0.05, **p ≤ 0.01, ***p < 0.001 and ****p < 0.0001 compared to the control group.

**Figure 3:**
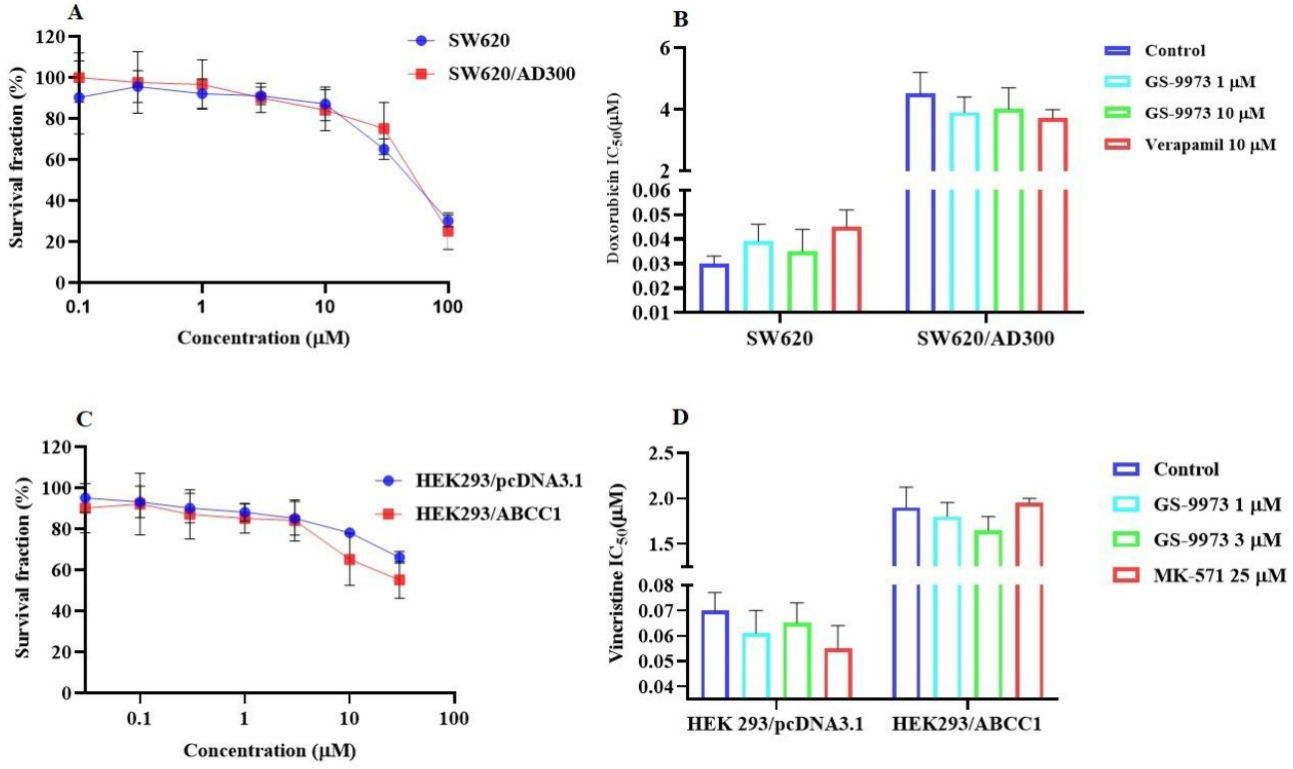
** The effect of GS-9973 in SW620 parental, ABCB1-overexpressing SW620/AD300 colon cancer cells and HEK293/pcDNA3.1 parental and HEK293/ABCC1 transfected cells. (A)** The survival fraction (%) for the SW620 parental and SW620/AD300 colon cancer cell lines were determined following incubation with 0-100 µM of GS-9973 for 72 h. **(B)** The IC50 values of doxorubicin in the presence of vehicle (Control), GS-9973 (1 or 10 µM) or verapamil (10 µM) for 72 h in SW620 parental and SW620/AD300 colon cancer cells. **(C)** The survival fraction (%) for the HEK293/pcDNA3.1 (empty DNA vector control) and HEK293/ABCC1 (transfected with the DNA coding for the ABCC1 transporter) cells were determined following incubation with 0-30 µM of GS-9973 for 72 h. **(D)** The IC50 values of vincristine in the presence of vehicle (Control), GS-9973 (1 or 3 µM) or MK-571 (25 µM) for 72 h in HEK293/pcDNA3.1 and HEK293/ABCC1 cells. The points with error bars represent the mean ± SD of independent determinations in triplicate. The figures are representative of three independent experiments.

The MTT assay data indicated that the nontoxic concentrations of GS-9973 (concentration at which 85% of cells survive) were 1 and 3 µM (Figure 1A, 2A, 3C) and 1 and 10 µM (Figure 3A). These concentrations of GS-9973 markedly increased the cytotoxicity of mitoxantrone (Figure 1B) and doxorubicin in the drug-resistant cell line, NCI-H460/MX20 (Figure 1C) and in the transfected resistant cell lines, HEK293/R482 (wild-type), HEK293/R482G and HEK293/R482T (2 variants) to mitoxantrone (Figure 2B) and doxorubicin (Figure 2C). The resistance-fold values were reversed in NCI-H460/MX20 cancer cells treated with mitoxantrone (from 73.3- to 13.9-fold resistance), and doxorubicin (155.7 - to 11.1-fold resistance) individually (Table 1) in the presence of 3 µM of GS-9973. Besides, 3 µM of GS-9973 also reversed the drug resistance to the anticancer drugs, mitoxantrone and doxorubicin in the cells transfected with ABCG2 (Table 2). The reversal activity of 3 µM of GS-9973 for the ABCG2 transporter was compared to the same concentration of Ko143 68. Moreover, GS-9973 did not change the efficacy of drugs, mitoxantrone and doxorubicin in the NCI-H460 parental non-resistant cell line. Also, GS-9973, at 3 µM, could not modify the efficacy of the anticancer drug, cisplatin, (not an ABCG2 substrate) 69 (Figure 1D and Figure 2D). Finally, 10 µM of GS-9973 did not remarkably reverse the drug resistance of 1) SW620/AD300 colon cancer cells, which have high expression of the ABCB1 transporter, to doxorubicin (Figure 3B), 2) 3 µM of GS-9973 did not modify the efficacy of the anticancer drug, vincristine in HEK293/ABCC1 cells, which overexpress the ABCC1 transporter (Figure 3D). Overall, our results show that GS-9973 reverses the anticancer drug resistance to mitoxantrone and doxorubicin in wild-type and mutant-type cancer cell lines that overexpress the ABCG2 transporter.

### GS-9973 significantly decreases the efflux of [^3^H]-mitoxantrone in NCI-H460/MX20 cancer cells

**Figure 4:**
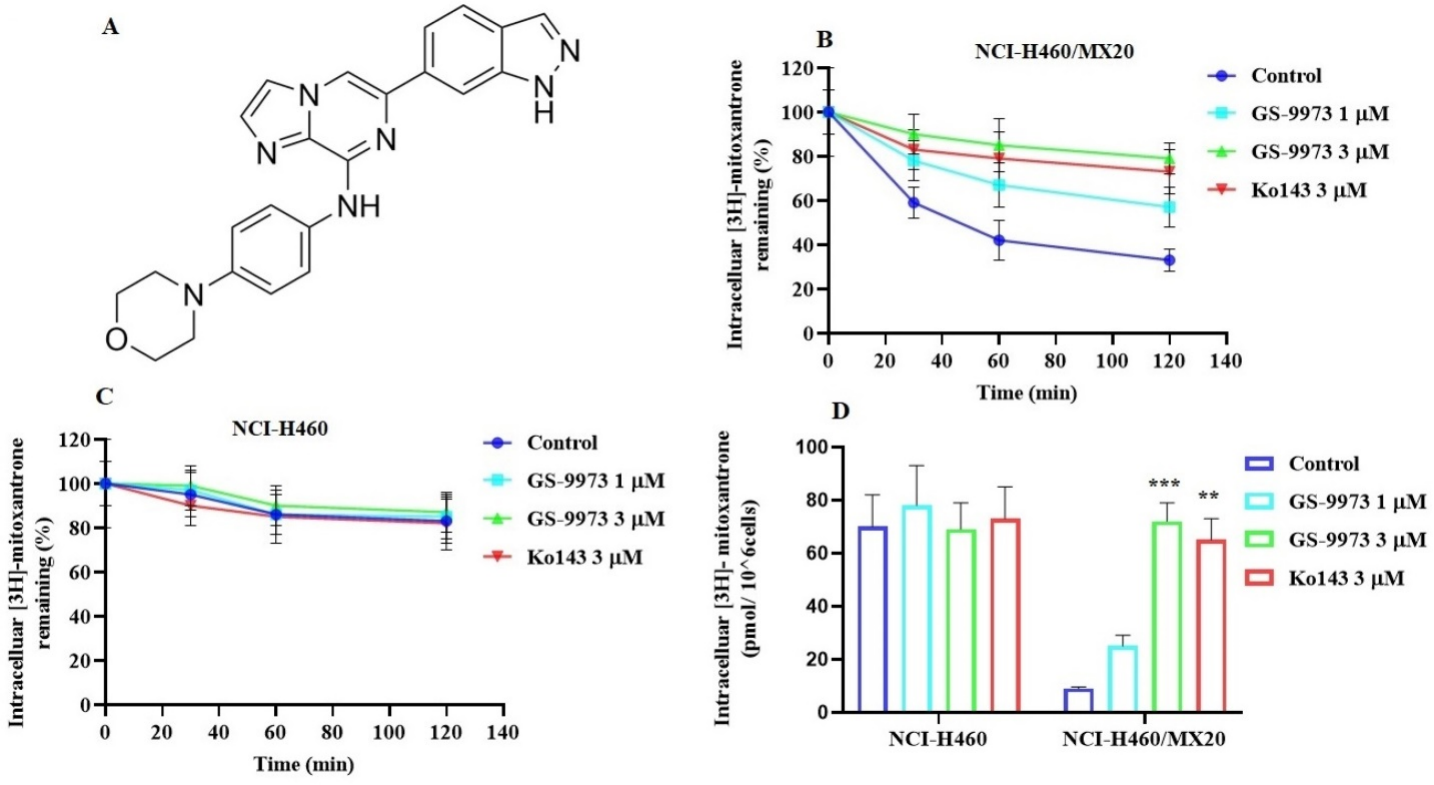
** (A)** The chemical structure of GS-9973. **(B)** The effect of the incubation of vehicle (Control), GS-9973 (1 or 3 µM) or Ko143 (3 µM) for 30, 60 or 120 min on the intracellular level of the ABCG2 transporter substrate, [^3^H]-mitoxantrone from NCI-H460/MX20 cancer cells overexpressing the ABCG2 transporter. **(C)** The effect of the incubation of vehicle (Control), GS-9973 (1 or 3 µM) or Ko143 (3 µM) for 30, 60 or 120 min on the intracellular level of the ABCG2 transporter substrate, [^3^H]-mitoxantrone from NCI-H460 parental cancer cells. **(D)** The effect of vehicle (Control), GS-9973 (1 or 3 µM) or Ko143 (3 µM) on the intracellular accumulation of [^3^H]-mitoxantrone in NCI-H460 and NCI-H460/MX20 cancer cells. The columns are the mean of triplicate determinations; the error bars represent the SD. **p ≤ 0.01 and ***p < 0.001 compared with control group.

To figure out the mechanism by which GS-9973 reverses drug resistance, the accumulation and efflux assay was accomplished using radioactive tritium labeled [^3^H]-mitoxantrone in the sensitive cell line, NCI-H460 and its mitoxantrone-resistant NSCLC cell line, NCI-H460/MX20. The accumulation of [^3^H]-mitoxantrone in the mitoxantrone-resistant NCI-H460/MX20 cancer cells was substantially low compared to the parental cancer cells. The accumulation of [^3^H]-mitoxantrone in the resistant cell line, NCI-H460/MX20 was significantly increased after incubation with 3 μM of GS-9973 compared to vehicle. The treatment of parental cells with 3 µM of GS-9973 has not shown any remarkable increase in the accumulation of [^3^H]-mitoxantrone (Figure 4D). These outcomes show that GS-9973 enhances the level of [^3^H]-mitoxantrone in NCI-H460/MX20 cells compared to vehicle.

To determine if the rise in the level of [^3^H]-mitoxantrone in MDR cells was due to GS-9973 preventing the ABCG2 transporter efflux activity, we measured [^3^H]-mitoxantrone levels in the drug-resistant cells with or without GS-9973 at various time points. In the NCI-H460/MX20 cancer cells, intracellular accumulation of [^3^H]-mitoxantrone were 59%, 42%, and 33% that of NCI-H460 parental cancer cell line, at 30, 60, and 120 min, respectively, in the absence of GS-9973 treatment (Figure 4B), proving that the efflux of [^3^H]-mitoxantrone was mediated by the ABCG2 transporter. The efflux of [^3^H]-mitoxantrone was significantly reduced with GS-9973 at a concentration of 3 µM thereby increasing the intracellular [^3^H]-mitoxantrone level (90% at 30 min, 85% at 60 min, and 79% at 120 min (Figure 4B) The proportion of the decrease in the efflux of [^3^H]-mitoxantrone by GS-9973 was comparable to that of 3 µM of Ko143. intracellular concentration of [^3^H]-mitoxantrone in the parental cancer cells was not notably changed by 3 µM of GS-9973 in comparison with the cells incubated with the vehicle (Figure 4C).

In conclusion, our results indicated that GS-9973 significantly enhanced the [^3^H]-mitoxantrone accumulation in NCI-H460/MX20 cancer cells by blocking the pumping out function of the ABCG2 transporter.

### The effect of GS-9973 on the expression of ABCG2

**Figure 5:**
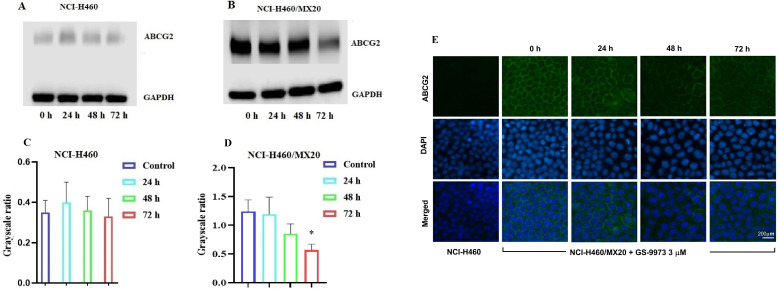
** The effect of GS-9973 on the expression of the ABCG2 protein.** Analysis of ABCG2 expression in **(A)** NCI-H460 and **(B)** NCI-H460/MX20 cancer cells after the cells were incubated with 6 µM of GS-9973 for 24, 48 and 72 h. Relative quantification of the effect of GS-9973 on ABCG2 in **(C)** NCI-H460 and **(D)** NCI-H460/MX20 cells. ABCG2 expression levels were normalized to GAPDH. Equal amounts of total cell lysates were employed for each sample and Western blot analysis was performed. *p ≤ 0.05 compared with control group. **(E)** The effect of GS-9973 on the expression and localization of ABCG2 using immunofluorescence. The effect of incubation of NCI-H460 and NCI-H460/MX20 cancer cells with 3 µM of GS-9973 for 0, 24, 48 and 72 h. The green color represents the presence of the ABCG2 transporter, and the blue color represents the nucleus.

**Figure 6:**
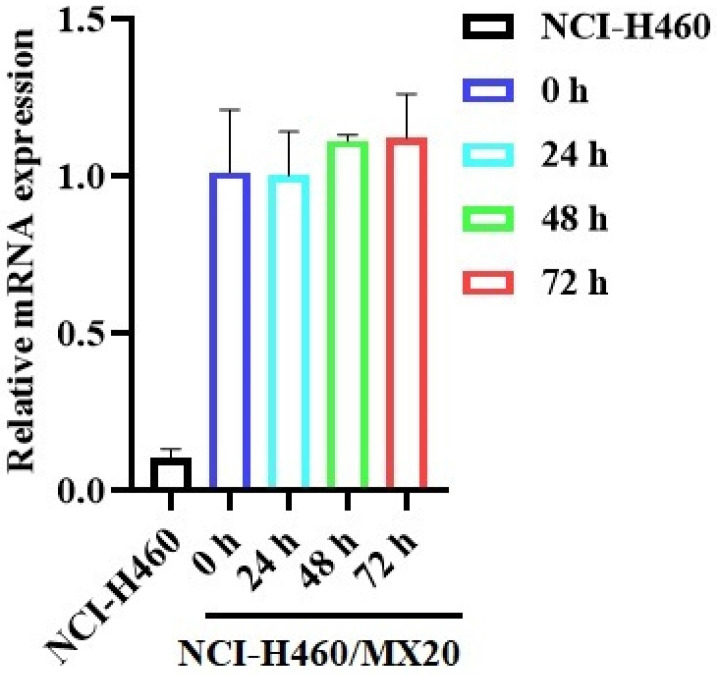
** The effect of GS-9973 on ABCG2 mRNA expression.** After incubation of NCI-H460 and NCI-H460/MX20 cancer cells with 3 µM of GS-9973 for 0, 24, 48 and 72 h, RT-PCR was conducted to determine the expression of ABCG2 mRNA.

The reversal effect of GS-9973 on the ABCG2-mediated MDR can be due to the inhibition of the efflux function of ABCG2 and/or the downregulation of the ABCG2 transporter expression. Therefore, Western blot analysis was performed to determine the GS-9973 mechanism on the ABCG2 protein expression in the parental and resistant cancer cells. The treatment of NCI-H460/MX20 cancer cells with 3 µM of GS-9973 for 72 h significantly inhibited the ABCG2 protein expression level compared to the untreated cells (Figure 5B and D). These results combine with the efflux of [^3^H]-mitoxantrone results, showed that the reversal action of GS-9973 in the ABCG2 overexpressed cancer cells was due to the inhibition of the ABCG2 protein expression and by blocking the transport function. The parental cell line, NCI-H460 has a very minimal expression of ABCG2 protein (Figure 5A and B) which further proves the selective action of GS-9973 on the ABCG2 overexpressing cell line. In addition, RT-PCR (quantitative real-time PCR) experiments indicated that the incubation of NCI-H460/MX20 cancer cells with 3 μM of GS-9973 for 72 h did not change the level of ABCG2 mRNA expression (Figure 6).

### The effect of GS-9973 on the cellular localization of the ABCG2 transporter protein

The reversal effect of GS-9973 in NCI-H460/MX20 cancer cells could be a change in the subcellular localization of ABCG2 from the cell membrane (i.e., the transporter would not be in the cell membrane, producing a decrease in drug efflux from the cells). Hence, an immunofluorescence assay was carried out to establish if GS-9973 affected the sub-cellular localization of the ABCG2 transporter from the cell surface to the cytoplasm. The incubation of the drug-resistant cells with 3 µM of GS-9973 did not have any effect on the subcellular ABCG2 transporter localization from the cell membrane to the cytosol (Figure 5E).

### GS-9973 stimulates the activity of ABCG2 ATPase

**Figure 7:**
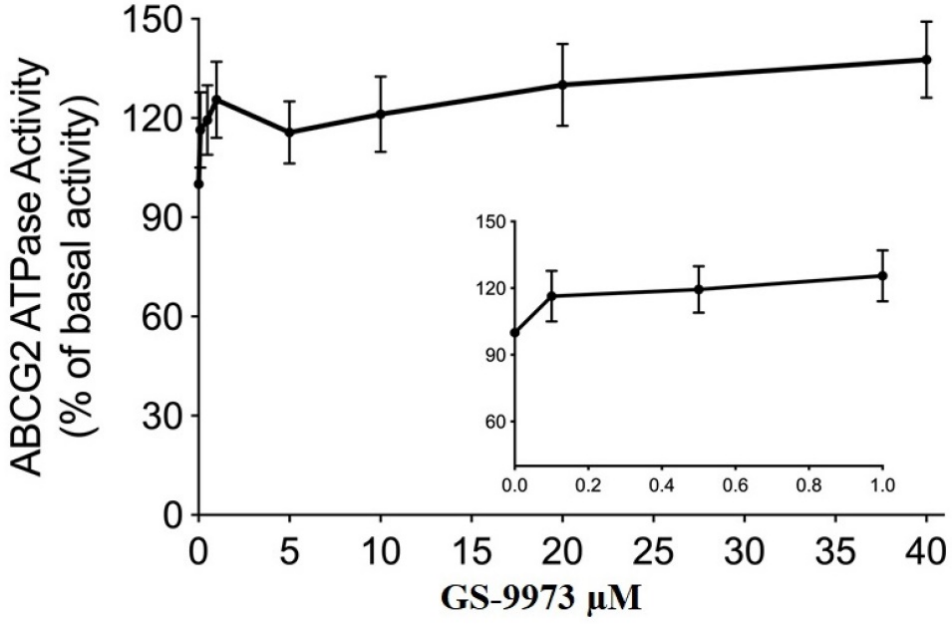
** GS-9973 stimulated the ATPase activity of ABCG2.** The graph illustrates the effect of 0-40 µM of GS-9973 on the ATPase activity of ABCG2.

The drug efflux function of the ABCG2 transporter is linked to ATP hydrolysis and can be stimulated or inhibited by ABCG2 substrates 70. To further assess the effect of GS-9973 on ABCG2 ATPase activity, we measured the ABCG2-mediated ATP hydrolysis after incubation with different concentrations of GS-9973 (0-40 μM). According to the result, GS-9973 showed dose-dependent stimulation of ABCG2 ATPase activity and it achieved a maximum of 137.7% of the basal activity of ABCG2 ATPase (Figure 7). The stimulation effect of GS-9973 attained 50% maximal (EC_50_) at 0.42 μM.

### The docking simulation of GS-9973 in the drug-binding pocket of the human ABCG2 protein

**Figure 8:**
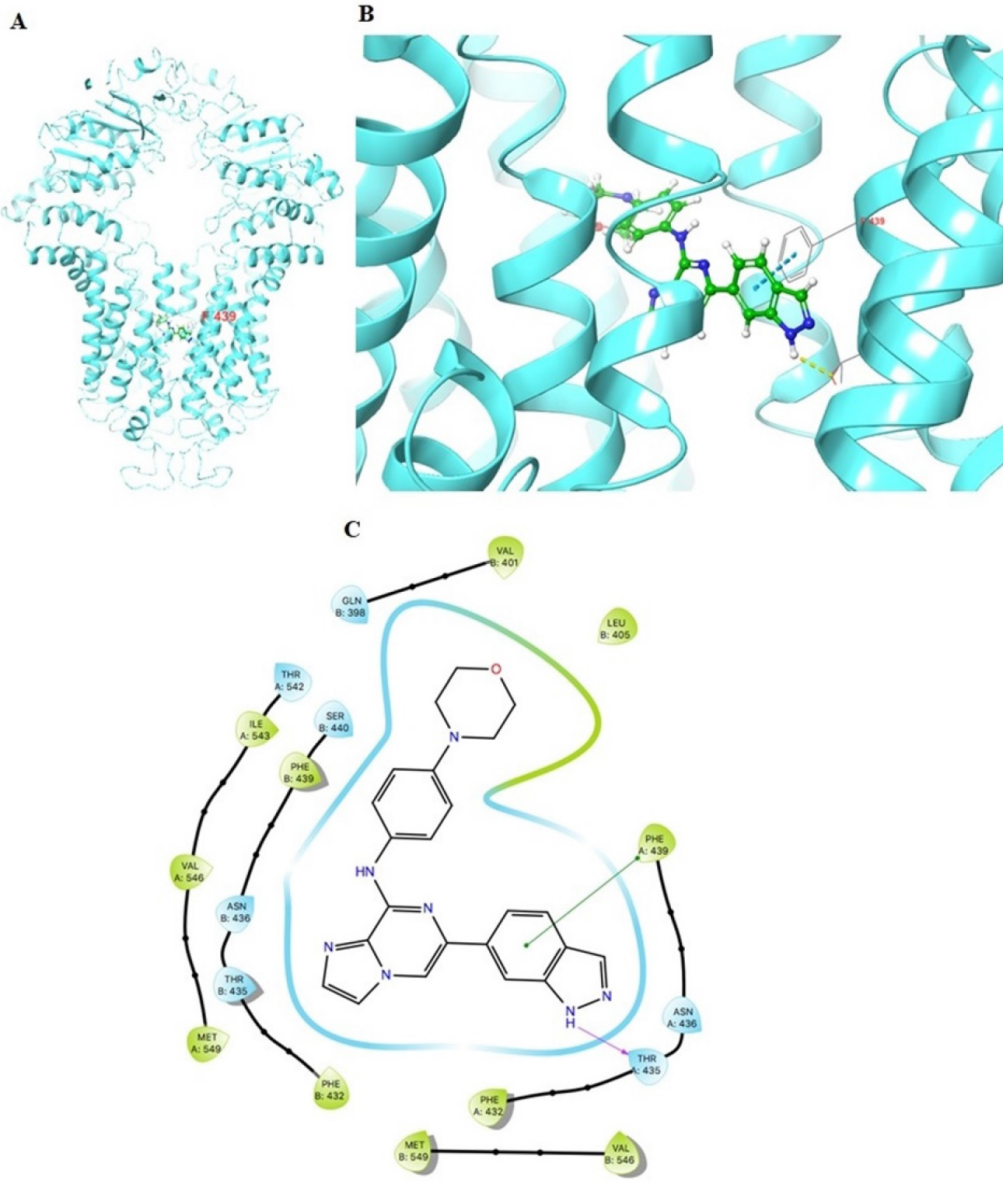
**Molecular interaction of GS-9973 with the human ABCG2 model. (A)** Whole ABCG2 protein with the docking site (highlighted with black box). **(B)** Docking pose of GS-9973 within the binding pocket of ABCG2. The protein is represented as sky blue colored ribbons. Amino acid residues are represented as follows: carbon in gray, hydrogen in white, nitrogen in blue and oxygen in red. The ligand is represented by the ball and stick model with carbon atoms are represented in green, oxygen in red nitrogen in blue and hydrogen in white. Blue dashes represent π-π stacking interaction, yellow dashes represent the hydrogen bonding. **(C)** 2-D ligand interaction between GS-9973 and ABCG2. Green indicates π-π interaction with amino acid residues within 5 Å of the ligand and the magenta arrow represents hydrogen bonding.

The previously established human ABCG2 cryo-EM structure model (PDB code: 6VXI) was employed for docking analysis. Induced-fit docking analysis was performed to stimulate interactions between GS-9973 and human ABCG2. The best-docked pose of GS-9973 had a Glide score of -10.226 kcal mol^-1^, which indicates a better binding affinity in the ABCG2 drug-binding pocket. Figure 8 shows the docking pose and interaction between GS-9973 and ABCG2 protein. It depicts π-π interaction between Phe439 and the phenyl adjacent to the imidazole ring and H- bonding is seen between the sidechain of Thr435 and nitrogen of imidazole ring of GS-9973.

### HPLC analysis

**Figure 9:**
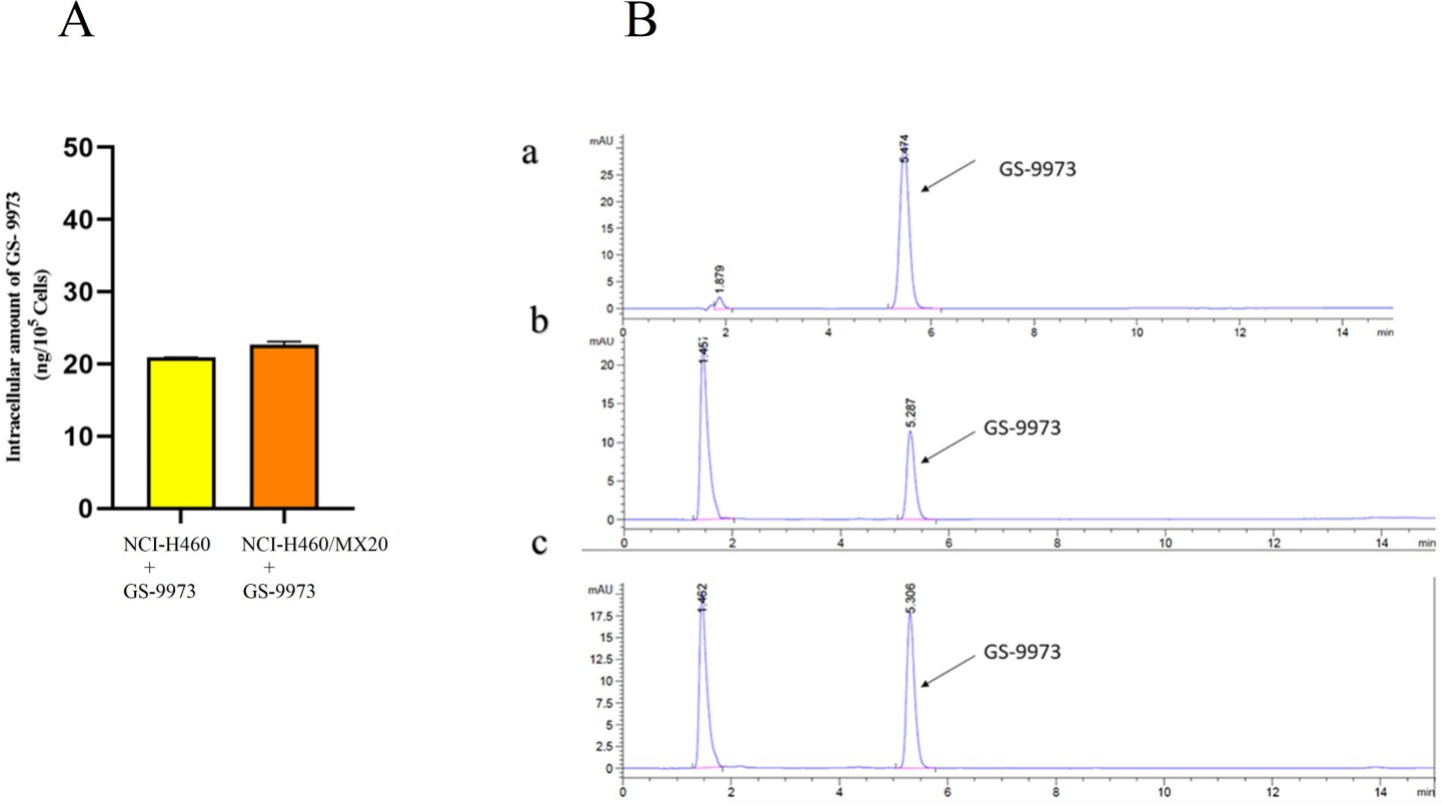
** (A)** The intracellular accumulation of GS-9973 in NCI-H460 and NCI-H460/MX20. Data are expressed as mean ± SD from three independent experiments. **(B)** HPLC trace at 254 nm for: a) GS-9973, b) Accumulation of GS-9973 in NCI-H460, c) Accumulation of GS-9973 in NCI-H460/MX20.

The retention time (t_R_) of GS-9973 was first determined. The t_R_ was found to be 5.3 min under the specified conditions (Figure 9B a). A standard curve was plotted. The samples collected from NCI-H460 and NCI-H460/MX20 were then injected, and the AUC was recorded (Figure 9B b and c), the drug concentration was calculated by using the equation from the standard curve. Each of the experiment was carried out in triplicates and the data were plotted using GraphPad prism. The HPLC data indicates that there is no remarkable difference in the intracellular accumulation of GS-9973 in NCI-H460 and NCI-H460/MX20 cells (Figure 9A).

## Discussion

GS-9973 is reported to be a potent and competitive inhibitor of Syk 49. Syk is involved in the signaling pathways in B cells, neutrophils, and mast cells 71, hence, GS-9973 has a promising role in various immune, cancer and inflammation-related disorders 72. It is currently under clinical trial for the treatment for CLL in combination with idelalisib, a PI3K inhibitor 73. In this study, experiments are conducted to figure out the MDR reversal effect of GS-9973 in the drug-resistant cell line, NCI-H460/MX20 that overexpress the ABCG2 transporter and mechanisms mediating the reversal activity of GS-9973 to overcome MDR. From the cytotoxicity studies, 1 and 3 μM of GS-9973 were used for the reversal experiments in the ABCG2 overexpressing cell line as the cell survival fraction was around 85% after 72 h incubation in both the NCI-H460 parental and resistant NCI-H460/MX20 cancer cells. As shown before, the anticancer efficacy of mitoxantrone and doxorubicin were notably decreased in the drug-resistant cancer cells, in comparison with the parental NCI-H460 cells.

The two concentrations, 1 or 3 µM of GS-9973 significantly magnified the anticancer efficacy (i.e., decreased drug resistance) of the ABCG2 substrates, mitoxantrone and doxorubicin in NCI-H460/MX20 cells, in comparison with the untreated cells. This data is consistent with the previous studies that demonstrated the activity of various anticancer drugs in the sensitive cancer cells. In addition, the reversal effect of GS-9973 in NCI-H460/MX20 cells was the same as that of 3 µM of Ko143, suggesting that GS-9973 has a significant reversal effect in ABCG2-overexpressing cells. GS-9973 has some advantages over Ko143, as at high concentrations, Ko143 can acts as an inhibitor of both ABCB1 abs ABCC1 74 which indicates the lack of selectivity towards ABCG2. Moreover, the efficacy of the anticancer drug, cisplatin was not altered significantly by 3 µM of GS-9973 in the parental and resistant cancer cells proving that cisplatin is not an ABCG2 specific substrate which is consistent with the previous studies.

The occurrence of resistance to the anticancer drugs, mitoxantrone and doxorubicin are presented in HEK293 transfected cells with either the R482G or R482T mutations but not in the parental HEK293 cells. GS-9973 remarkably enhanced the efficacy of mitoxantrone and doxorubicin in these ABCG2 transfected cell lines. It is established that the high expression of the ABCG2 gene in the transfected HEK293 cells is the only mechanism that confers the resistance to the chemotherapeutic drugs, mitoxantrone and doxorubicin, the results suggest that reversal efficacy of GS-9973 is primarily to its interaction with the ABCG2 transporter. Previous studies have shown that the wild and mutant forms of ABCG2 transporter are differentially affected by ABCG2 substrates. For example, wild type ABCG2 (R2) transporters are more sensitive to anthracycline but resistant to methotrexate, whereas the mutant forms, G2 and T7, of the ABCG2 transporter are more resistant to anthracyclines, but not to methotrexate 75,76. It has been reported that many compounds vary in specificity regarding their inhibition of the ABCG2 transporter. For example, novobiocin only inhibits wild-type but GS-9973 inhibits both the wild-type and mutant forms of ABCG2 77.

To determine whether the ABCG2 reversal activity of GS-9973 was selective, a reversal study was performed in the SW620/AD300 and HEK293/ABCC1 cells which overexpress ABCB1 and ABCC1 transporter, respectively. Our results indicated that GS-9973, at 3 or 10 μM, did not affect the efficacy of vincristine and doxorubicin in these cells. Overall, our results suggest that GS-9973 is selectively interacting with the ABCG2 transporter. The experiments were performed to establish the mechanism of ABCG2 reversal activity by GS-9973 to the substrate drugs, mitoxantrone and doxorubicin.

To evaluate the reversal mechanism of GS-9973, drug accumulation and efflux assays were carried out using the radioactive [^3^H]-mitoxantrone in the sensitive, H460 and H460/MX20 (drug-resistant) cells. The intracellular level of [^3^H]-mitoxantrone in the resistant cancer cells, which overexpress the ABCG2 transporter, was decreased remarkably compared to that of the parental, drug-sensitive, NCI-H460 cells. In addition, GS-9973 showed a significant increase in the intracellular accumulation of [^3^H]-mitoxantrone in the MDR NCI-H460/MX20 cell line in a time-dependent manner but not in the parental NCI-H460 cancer cells. This increase in mitoxantrone accumulation could result from either GS-9973 blocking the pump out action of the ABCG2 transporter and/or increasing the cellular uptake of mitoxantrone. Our results indicated that GS-9973 caused a remarkable, time-dependent rise in the levels of [^3^H]-mitoxantrone by blocking the ABCG2 transporter's efflux activity. However, another mechanism by which GS-9973 can increase the efficacy of certain anticancer drugs that are ABCG2 substrates is by decreasing the expression of the ABCG2 protein. Based on the [^3^H]-mitoxantrone results, Western blotting and PCR experiments were carried out to figure out the reversal effect of GS-9973 on the *in vitro* expression of the ABCG2 transporter protein in the MDR NCI-H460/MX20 cancer cells. The data demonstrated that the treatment of NCI-H460/MX20 cells with 3 µM of GS-9973 for 72 h notably downregulates the ABCG2 protein compared to control but no change in ABCG2 expression at mRNA level. These results suggested that the downregulation of ABCG2 by GS-9973 occurred only at the translational level probably due to post-translational modification. Therefore, in this study, it is proven that the increase in the anticancer efficacy of substrate drugs, mitoxantrone and doxorubicin in the drug-resistant cells results from a downregulation of the ABCG2 transporter protein and by inhibiting the transporter function. It is also possible that GS-9973 could change the localization of the ABCG2 protein from the cell membrane, thus decreasing the number of ABCG2 transporter available for drug efflux. As a result, *in vitro* immunofluorescent experiment was conducted to discover the effect of GS-9973 on the subcellular localization of the ABCG2 protein from the cell membrane to the cytosol and the result suggested GS-9973 could not remarkably modify the location of the ABCG2 protein in NCI-H460/MX20, compared to the vehicle. However, we cannot eliminate the possibility that longer incubation times with GS-9973 may have significantly altered the cellular distribution of the ABCG2 transporter.

The efflux function of the ABCG2 transporter is coupled to ATP hydrolysis, the drug can act by inhibiting the ATPase or by stimulating the ATPase, we demonstrated the *in vitro* activity of GS-9973 on the hydrolysis of ATP by the ATPase domain of the ABCG2 transporter. Our results showed that GS-9973 caused a moderate concentration-dependent rise in ATPase activity which could be due to the interaction with the drug-substrate binding site present in the transmembrane domain and competitively blocking the efflux of the ABCG2 transporter substrates. Combining this data with other results, suggests that GS-9973 may interact with the transporter at the drug-binding site, hindering the binding and extrusion of other ABCG2 substrates, thereby sensitize ABCG2-overexpressing cell lines to mitoxantrone and doxorubicin.

To evaluate the mechanism of GS-9973, we performed molecular docking analysis to study the interaction of GS-9973 with the ABCG2 transporter, using a human ABCG2 model. The docking data proves that GS-9973 interacts with the drug-substrate binding pocket in the human homology model of the ABCG2 transporter and prevents the binding of other substrates to the transporter, thereby preventing the efflux of substrates such as mitoxantrone.

As GS-9973 was identified to interact with the ABCG2 transporter, HPLC analysis was carried out to prove that the overexpression of the ABCG2 transporter did not confer resistance to GS-9973. HPLC analysis was conducted to determine the intracellular accumulation of GS-9973 in NCI-H460 cells and in the drug-resistant NCI-H460/MX20 cells. The results show that the intracellular concentration of GS-9973 is not significantly altered in both parental and resistant cell lines and hence, we hypothesize that the drug-binding causes structural changes and inhibits the efflux of other substrates which warrants future investigation.

In conclusion, this study presents the importance of GS-9973 as a modulator of the ABCG2 transporter. In cancer patients, a combination of GS-9973 and ABCG2 substrate drugs is a beneficial treatment option for cells with high ABCG2 expression. Finally, it is important to note that the *in vitro* results are insufficient for determining the potential clinical use of a drug and therefore, further data must be obtained using *in vivo* animal models to determine the efficacy and safety of GS-9973.

## Figures and Tables

**Table 1:** The effect of GS-9973 on reversal of ABCG2 mediated MDR in the drug-resistant cell lines

Cell lines	NCIH460		NCIH460/MX20	
Compounds	IC_50_ ±SD (µM)	FR	IC_50_ ±SD (µM)	FR
**Mitoxantrone**	0.115±0.013	[1.0]	7.352±0.843	[73.3]
+ GS-9973 (1 µM)	0.123±0.021	[1.1]	3.612±0.433	[36.1]
+ GS-9973 (3 µM)	0.151±0.025	[1.3]	1.395±0.051	[13.9]
+Ko 143 (3 µM)	0.101±0.015	[0.9]	1.251±0.015	[12.5]
**Doxorubicin**	0.108±0.015	[1.0]	15.579±1.835	[155.7]
+ GS-9973 (1 µM)	0.115±0.026	[1.2]	4.363±0.523	[43.6]
+ GS-9973 (3 µM)	0.135±0.02	[1.7]	1.119±2.3	[11.1]
+Ko 143 (3 µM)	0.09±0.011	[0.7]	1.982±0.277	[19.9]
**Cisplatin**	144.959±22.426	[1.0]	164.845±23.119	[1.3]
+ GS-9973 (1 µM)	149.259±23.131	[1.1]	143.985±27.406	[1.1]
+ GS-9973 (3 µM)	153.98±19.123	[1.0]	156.876±21.99	[1.1]
+Ko 143 (3 µM)	156.51±15.951	[1.0]	165.785±19.335	[1.3]

µM-Micromole. Values in tables indicate least three independent experiments performed in triplicates. IC_50_: concentration of the drug that is required for inhibition of cell survival by 50% (mean ± SD). FR: Resistance fold was calculated by dividing the IC_50_ values of anticancer drugs in drug-resistant cells in the presence or absence of inhibitor by the IC_50_ of parental cells without inhibitor.

**Table 2:** The effect of GS-9973 on reversal of ABCG2 mediated MDR in the transfected cell lines

Cell lines	HEK293/pcDNA3.1		HEK293/R2		HEK293/G2		HEK293/T7	
Compounds	IC_50_ ±SD (µM)	FR	IC_50_ ±SD (µM)	FR	IC_50_ ±SD (µM)	FR	IC_50_ ±SD (µM)	FR
**Mitoxantrone**	0.07 ± 0.009	[1.0]	1.98 ± 0.27	[28.2]	1.57 ± 0.16	[22.5]	2.3 ± 0.57	[32.8]
+ GS-9973 (1µM)	0.06 ± 0.006	[1.25]	0.52 ± 0.09	[7.3]	0.75 ±0.19	[10.7]	0.957 ± 0.14	[12.7]
+GS-9973 (3 µM)	0.05 ± 0.01	[0.94]	0.09 ± 0.03	[1.3]	0.05 ± 0.009	[0.7]	0.33 ± 0.06	[4.3]
+Ko143 (3 µM)	0.06 ± 0.02	[1.26]	0.08± 0.01	[1.1]	0.07 ± 0.01	[1.0]	0.29 ± 0.05	[4.1]
**Doxorubicin**	0.03± 0.004	[1.0]	1.33 ± 0.11	[43.3]	1.56 ± 0.16	[52.2]	1.27 ± 0.23	[40.1]
+GS-9973 (1 µM)	0.035 ± 0.007	[0.8]	0.85 ± 0.157	[18.3]	0.76 ± 0.05	[9.6]	0.95 ± 0.07	[19.0]
+GS-9973 (3 µM)	0.03 ± 0.01	[0.9]	0.15 ± 0.07	[5.0]	0.12 ± 0.02	[4.1]	0.16 ± 0.02	[5.3]
+Ko143 (3 µM)	0.031 ± 0.006	[1.0]	0.19 ± 0.04	[6.3]	0.11 ± 0.02	[3.6]	0.13 ± 0.02	[4.3]
**Cisplatin**	20.33 ± 3.66	[1.0]	12.33 ± 2.22	[0.9]	17.348 ± 2.57	[0.9]	23.33 ± 3.36	[1.1]
+GS-9973 (1 µM)	22.99 ± 3.55	[1.1]	13.73 ± 2.24	[0.9]	15.99 ± 2.56	[1.1]	24.12± 4.63	[1.1]
+GS-9973 (3 µM)	25.35 ± 5.12	[1.1]	15.25± 3.61	[1.0]	17.55 ± 2.11	[0.9]	25.55 ± 4.65	[1.0]
+Ko143 (3 µM)	23.95 ± 3.39	[1.1]	14.52 ± 1.57	[1.1]	14.33 ±2.33	[1.0]	19.79 ± 3.30	[1.0]

µM-Micromole. Values in tables indicate least three independent experiments performed in triplicates. IC_50_: concentration of the drug that is required for inhibition of cell survival by 50% (mean ± SD). FR: Resistance fold was calculated by dividing the IC_50_ values of anticancer drugs in transfected cells in the presence or absence of inhibitor by the IC_50_ of parental cells without inhibitor.
